# Measurement of Neuropsychiatric Symptoms in Clinical Trials Targeting Alzheimer’s Disease and Related Disorders

**DOI:** 10.3390/ph3082387

**Published:** 2010-07-26

**Authors:** Renaud David, Emmanuel Mulin, Patrick Mallea, Philippe H. Robert

**Affiliations:** 1 Centre Mémoire de Ressources et de Recherche/Hôpital de Cimiez, 4 av Reine Victoria, 06000 Nice, France; E-Mails: robert.ph@chu-nice.fr (P.H.R.); mulin.e@chu-nice.fr (E.M.); 2 Centre d’Innovation et d’Usage en Santé (CIU-S) / Hôpital de Cimiez, 4 av Reine Victoria, 06000 Nice, France; E-Mail: mallea.p@chu-nice.fr (P.M.)

**Keywords:** neuropsychiatric symptoms, assessment tools, Alzheimer’s disease, clinical trials, actigraphy

## Abstract

Behavioral and psychological symptoms (BPSD) are now known to be frequently associated to cognitive and functional decline in Alzheimer‘s disease and related disorders. They are present since the early stages of the disease and have negative impact on the disease process. BPSD assessment is crucial in clinical practice and also in future clinical trials targeting disease-modifying therapies for dementia. In this article, we will first review current assessment tools for BPSD, mainly global and domain-specific scales, and new assessment methods, currently available or in development, including new scales, diagnostic criteria and new technologies such as ambulatory actigraphy.

## 1. Introduction

Beside cognitive dysfunction and loss of autonomy, neuropsychiatric symptoms (NPS) also called behavioral and psychological symptoms (BPSD) are frequently observed during the evolution of Alzheimer’s disease (AD) and are now proposed as a major component of the dementia syndrome.

According to the majority of epidemiological studies, apathy, depression, and anxiety are the most common NPS in AD [1,2] (Table 1). 

**Table 1:** Frequency of BPSD evaluated with the NPI in three European studies. MAASBED: Maastricht Study of Behavior in Dementia, REAL: Réseaux Alzheimer Français, EADC: European Alzheimer Disease Consortium.

	TotalN = 836 (%)	MAA SB EDMMSE* 15-28n = 199 (%)	REAL-FRMMSE 11-20n = 235 (%)	REAL-FRMMSE 21-30n = 244 (%)	EADCMMSE 4-28n = 138 (%)
**Apathy**	55.5	59.3	63.5	47.9	48.9
**Depression**	44.9	57.3	42.7	36.9	45.3
**Anxiety**	42.0	39.2	46.3	44.3	33.8
**Agitation**	35.0	28.6	44.3	32.8	30.9
**Irritability**	30.6	39.7	25.0	28.3	31.7
**Aberrant motor behavior**	24.7	34.7	29.8	14.7	18.7
**Delusion**	22.0	34.7	24.7	10.2	19.4
**Appetite**	21.4	24.6	24.3	20.5	12.9
**Sleep**	14.3	18.1	12.9	13.5	12.9
**Disinhibition**	12.4	12.6	13.3	10.2	14.4
**Hallucinations**	8.5	13.1	7.8	5.7	7.9
**Euphoria**	6.8	7.0	9.8	4.5	5.0

There is a growing interest in these symptoms as they are present since the early stage of the disease, constitute a marker of disease progression, are responsible for a large share of the suffering of patients and caregivers, and strongly determine the patient’s lifestyle and management. Behavioral changes are not only important at a symptomatic level but could be a key feature for the future disease-modifying therapies. The aim of such therapies is to modify durably patients’ symptomatology and to delay the progression to the most severe stage of the disease. As BPSD become on average more frequent with the disease progression, their assessment in the course of a trial is particularly relevant on the effect of disease-modifying agents. In this context it is important to improve the quality of the current clinical instruments. This article is structured in order to review current assessment tools commonly used in clinical trials, to present more recent clinical evaluation methods, including diagnostic criteria and improvements of current tools, and to highlight the importance of new technologies in this field.

## 2. Current Assessment Tools

Global scales for behavioral disorders have been previously examined by Perrault and Forester [3,4], and are presented in Table 2. 

The most frequently used in clinical trials is the neuropsychiatric Inventory (NPI) [5]. Most of the studies used the NPI total score representing the sum of the 12 neuropsychiatric “frequency x severity score”. The NPI total score is inappropriate since it does not provide information on specific NPS, which have various symptoms and etiologies [14]. Thus, the use of NPI sub syndromes [15] or single item score is preferable. Despite its many advantages, the NPI has several limitations, including lack of clinician judgement, limited depth of items, and lack of specificity to all stages of dementia. In addition results also indicated high standard deviations of measurement within trials reflecting important variability. Recently clarification of administration and scoring rules [16] has been proposed. In addition to the most common global assessment scales, other global instruments are also available such as the Frontotemporal Behavioural Scale (FBS) [17] or the Frontal Behavioural Inventory (FBI), have also been proposed to assess behavioral disturbances. This last scale was initially proposed to evaluate behavioral symptoms in frontotemporal dementia. 24 items are evaluated including positive and negative symptoms [18]. However, limited numbers of studies have used this scale. 

Other scales are available to assess a particular type of challenging behaviour in more depth. Examples of these scales are the Cohen-Mansfield Agitation Inventory (CMAI) [19], the Cornell Scale for Depression in Dementia [20], the Geriatric Depression Scale [21], the Apathy Evaluation Scale (AES) [22], the Apathy Scale (AS) [23], the Apathy Inventory (AI) [24], the Lille Apathy Rating Scale (LARS) [25]. These instruments have all been validated in several settings, and translations are available in most of the western languages. The apathy and depression instruments are particularly important in the early, mild stage of the disease. 

**Table 2:** Brief description of global behavioral assessment batteries.

Scale	Brief description
**Neuropsychiatric Inventory (NPI [5])**	12 domains, consisting of 7 to 9 items within each domain, based on caregiver interview. Frequency and severity over the past month are rated. This scale has limited data regarding responsiveness to change
**Brief Psychiatric Rating Scale (BPRS [6])**	This scale, originally developed to evaluate response to pharmacologic treatment in psychiatric disorders [7], is a 16-item rated on severity assessment method administered to patient. It shows limited utility as an outcome measurement scale in drugs trial for AD [3].
**AD Assessment Scale-non cognitive (ADAS-noncog [8])**	This 10-item scale, developed to measure change in AD patients following pharmacologic treatment, doesn’t include all behavioral symptoms that can be observed in AD and doesn’t appear to be the most appropriate tool to assess accurately behavioral change in clinical trials
**Behavioral Pathology in AD Rating Scale (BEHAVE-AD [9])**	This 26-item scale, based on caregiver interview, is more specific for psychotic disorders of demented patients and was designed for pharmacologic trials [9,10].
**Relative’s Assessment of Global Symptomatology (RAGS [11])**	This is a 21-item self administered scale for psychiatric and behavioral symptoms of community dwelling elderly
**Consortium to Establish a Registry for AD behavior Rating Scale for Dementia (C-BRSD)**	This scale, based on caregiver interview, is designed to assess severity of behavior over the past month (46- or 48-items) [12].
**Dementia Behavior Disturbance Scale (DBD [13])**	This 28-item scale, rated by caregiver on frequency over the past week

## 3. New Scales and Diagnostic Criteria

Due to the limitations of the previously described NPI, a revised version, the NIP-C [26], has been recently proposed, in collaboration with the original developer of the NPI. The first aim of the NPI-C is to provide additional content so that individual, expanded domains can be used as needed for studies examining specific NPS changes rather than using different stand-alone instruments (e.g., CMAI) in addition to the traditional NPI. The expanded items also cover a wider spectrum of dementia, from mild to severe. The second purpose of the NPI-C is to provide a methodology for a “clinician judgment” rating. The NPI-C makes use of the patient and caregiver interview. The clinician rating then uses whatever additional information he/she needs to provide a final rating.

Another important point is the use of diagnostic criteria for BPSD. Scales are fundamental for the quantitative assessment but, in the same time, research in the field of BPSD is hampered by the lack of generally accepted criteria for these symptoms. Diagnostic criteria for psychosis [27] and depression [28] has been developed for this purpose and more recently, diagnostic criteria for apathy (Table 3) have been proposed [29]. These criteria have been validated in several neuropsychiatric diseases [30]; their use will simplify the selection of patients with or without apathy in clinical and therapeutic researches.

**Table 3:** Diagnostic Criteria for Apathy [21].

For a diagnosis of apathy the patient should fulfil criteria **A**, **B**, **C** and **D** **A. - Loss of, or diminished, motivation in comparison to the patient’s previous level of functioning and which is not consistent with his/her age or culture. These changes in motivation may be reported by the patient or by the observations of others.** **B. - Presence of at least one symptom in at least two of the three following domains for a period of at least four weeks and present most of the time** **Domain B1- Behaviour:** **Loss of, or diminished, goal-directed behaviour** as evidenced by at least one of the following: - Initiation symptom: loss of self-initiated behaviour (for example: starting conversation, doing basic tasks of day-to-day living, seeking social activities, communicating choices); - Responsiveness symptom: loss of environment-stimulated behaviour (for example: responding to conversation, participating in social activities). **Domain B2 - Cognition:** **Loss of, or diminished, goal-directed cognitive activity** as evidenced by at least one of the following: - Initiation symptom: loss of spontaneous ideas and curiosity for routine and new events (e.g., challenging tasks, recent news, social opportunities, personal/family and social affairs); - Responsiveness symptom: loss of environment-stimulated ideas and curiosity for routine and new events (e.g., in the person’s residence, neighbourhood or community). **Domain B3 - Emotion:** **Loss of, or diminished, emotion** as evidenced by at least one of the following: - Initiation symptom: loss of spontaneous emotion, observed or self-reported (e.g., subjective feeling of weak or absent emotions, or observation by others of a blunted affect); - Responsiveness symptom: loss of emotional responsiveness to positive or negative stimuli or events (e.g., observer reports of unchanging affect or of little emotional reaction to exciting events, personal loss, serious illness, emotional-laden news). **C. - The symptoms in criteria A and B cause clinically significant impairment in personal, social, occupational, or other important areas of functioning.** **D. - The symptoms in criteria A and B are not exclusively explained by or due to any of the following: physical disabilities (e.g., blindness and loss of hearing), motor disabilities, diminished level of consciousness or the direct physiological effects of a substance (e.g., drug abuse, medication)**

## 4. New Technologies for the Assessment of BPSD

Gerontechnologies have been proposed in several studies as a new approach for the evaluation of behavioral symptoms, and could provide in the future more objective evaluation tools in clinical trials. New technologies, currently available or in development, are presented in the following section, with a main focus on actigraphy.

### 4.1. Actigraphy

Ambulatory sensors for human motor activity have been initially proposed as an objective method to evaluate sleep patterns [31] and psychiatric symptoms [32]. Actigraphy, consisting of a small relatively unobtrusive device containing a piezoelectric accelerometer, is frequently used to monitor human motor activity and has been proposed as an indirect method to evaluate different disorders including mainly sleep/wake [33,34], but also Attention Deficit/Hyperactivity [35] and Periodic Limb Movement Disorder [36]. For the determination of sleep/wake patterns, the actigraph is usually worn on the non-dominant wrist over several consecutive 24 h-periods. Using specific algorithms [37,38], sleep/wake data are derived from patterns of activity and inactivity. In the field of dementia, actigraphy is widely used as an objective assessment method to evaluate AD patients’ fragmented sleep [33,34]. Because the devices are generally well-tolerated, data can be collected for multiple nights unlike polysomnography, the gold standard to evaluate sleep disturbances, that requires patient’s cooperation typically for overnight in-hospital stays (usually for one or at most two nights), takes considerable technician time to set up and score and is likely more disturbing for AD patients than less obstructive devices such as wrist-worn actigraphs. In Alzheimer’s disease, actigraphy has also been used in the evaluation of several BPSD such as agitation [39,40,41] or characterization of depression [42,43,44].

Apathy is the most frequent BPSD and is a marker of conversion to AD. It has been previously shown that evaluation of patient’s motor activity using ambulatory actigraphy could be an indirect marker of apathy in traumatic brain injuries [45]. More recently, similar results have been obtained in two studies with AD patients. In the first study, thirty AD subjects and fifteen healthy controls wore a wrist-actigraphy (Actiwatch-L) over 75 minutes during a classical neuropsychological and behavioral examination (Table 4) [46]. 

AD patients were divided into two subgroups (with and without apathy) according to the Apathy Inventory (AI) [47]. As shown, AD patients with apathy had significantly lower mean motor activity than AD patients without apathy. In addition, all AD patients (with and without apathy) had lower mean motor activity than healthy controls. However, it must be underlined that actigraphy maybe inadequate in patients with comorbid mobility problems (Parkinsonism or arthritis) as they may enhance or decrease levels of motor activity. Thus, in the majority of actigraphic studies, presence of mobility problems represents a non-inclusion criterion for patients. In the second study, ninety three AD outpatients wore a wrist-actigraphy (MotionLogger) during seven consecutive days (24-hour periods) (Table 5) [48].

Patients were divided into two subgroups according to the apathy sub score of the NPI (patients with NPI-apathy sub-score greater than four were considered apathetic). Similarly, AD patients with apathy had significantly lower daytime mean motor activity (dMMA) while nighttime mean motor activity (nMMA) did not significantly differ between the two subgroups.

**Table 4:** 75 mn - Actigraphic parameters (mean ± SD) for AD patients (with and without apathy) and for a control group. Comparison between AD patients without apathy *vs.* AD patients with apathy (Mann-Whitney U-Test: p < 0.05 *, p < 0.01 **) and AD patients without apathy *vs.* Controls (Mann-Whitney U-Test; p < 0.05 ^†^, p < 0.01 ^††^). Mean motor activity in arbitrary actigraph units.

	Controls (n = 15)	AD without apathy (n = 17)	AD with apathy (n = 15)
**Sex ratio (M/F)**	0.60 ± 0.51	0.29 ± 0.47	0.27 ± 0.46
**Age (yrs)**	73.13 ± 6.01	78.65 ± 7.36†	80.20 ± 4.96
**MMSE**	30.00 ± 0.00	22.59 ± 2.72†	20.40 ± 3.16
**MADRS**	3.33 ± 3.08	5.29 ± 4.48	4.73 ± 4.93
**AI total score (caregiver)**	-	0.62 ± 1.19	13.69 ± 6.45**
**AI total score (patient)**	0.07 ± 0.27	0.00 ± 0.00	6.93 ± 8.42**
**AI total score (clinician)**	0.08 ± 0.29	2.40 ± 1.68	13.13 ± 4.75**
**Mean Motor Activity (MMA)**	43.93 ± 22.59	28.88 ± 18.27††	10.30 ± 10.98**

**Table 5:** Seven-day actigraphic parameters (mean ± SD) for AD patients (with and without apathy. Actigraphic parameters for the two groups and comparison between AD patients without apathy *vs.* AD patients with apathy (*t*-test: p < 0.05*, p < 0.01**); nMMA= nighttime mean motor activity, dMMA = daytime mean motor activity (arbitrary actigraph units).

	AD without apathy (n = 57)		AD with apathy (n = 36)	
**Sex ratio (M/F)**	0.4 ± 0.5		0.5 ± 0.5	
**Age**	75.5 ± 9.4		78.2 ± 5.3	
**MMSE**	21.9 ± 4.1		20.8 ± 5.0	
**NPI-apathy**	0.4 ± 0.9		6.7 ± 2.5**	
**nMMA**	25.3 ± 11.3		26.9 ± 13.7	
**dMMA**	175.9 ± 26.5		154.8 ± 28.7**	

### 4.2. Others Technologies

Others technologies could also show interest for a more objective evaluation of BPSD, such as intra-red sensors, video detection, computerized tests, tracking technologies. Automated surveillance system based on passive infra-red sensors has been developed [49] and shown ability to identify correctly 89% sequences of movement, in comparison with manual analysis.

Reaction time task computerized test has been proposed for the evaluation of apathy in mild cognitive impairment and AD patients. Patients with apathy had significantly higher reaction times than patients without apathy (p < 0.05) [50]. 

Geolocalization methods, using tracking technologies such as global positioning system (GPS), could also be promising tools for the assessment of mobility behavior [51] and BPSD such as aberrant motor behavior (wandering, run away). However scientific evidences are not yet available in this field.

## 5. Discussion

Evaluation of “decline” in demented syndromes may refer to different domains: cognitive decline, behavioral and psychological symptoms, functional decline, and loss of quality of life. Specific evaluation instruments, currently available for each of these domains, are particularly important to assess treatment efficacy in dementia. In theory, treatments should improve all of these domains, but improvement of cognitive function has been until now the main target to assess anti-dementia drug efficacy. Thus, since BPSD are a major component of the dementia syndrome, there is a need also for assessment methods able to evaluate drug’s efficacy on BPSD because BPSD are key indicators of the disease progression. 

This article tried to present the different available options to assess BPSD. Each of the presented assessment methods has strengths and weaknesses. The first one refers to current assessment tools, including global and specific scales, which use input from either the patient and/or the caregiver. In this case, evaluation is usually easy to realize but remains partly subjective and lacks of sensitivity. Many different scales for BPSD are currently available. Thus, conflicting results have been reported among drug trials using different assessment scales [52]. The second option is to use new instruments resulting from improvement of current scales (NPI-C) or development of diagnostic criteria (diagnostic criteria of apathy). These new instruments are probably more powerful since their measures incorporate a clinician judgment. In this line, the recent NPI-C was partly developed to provide a single versatile rating method for BPSD that can be used in a range of clinical studies. It could limit in this way the risk of conflicting results observed in clinical trials targeting specific BPSD when different assessment tools are used. However, the NPI-C and the diagnostic criteria of apathy, presented in this article, have been recently developed and data concerning their interest and reliability for clinical trials, and most particularly drugs trials, are lacking.

Finally, the third option refers to new technologies such as actigraphy. Actigraphy has been used in human motor activity monitoring for more than two decades and provides a simple and objective assessment method for several neuropsychiatric symptoms. However, frequent comorbidities associated with older age, such as mobility problems and chronic pain, are known to potentially interact with actigraphic-assessed levels of motor activity and thus may bias the indirect assessment of a specific targeted neuropsychiatric symptom. In the field of AD, only several authors have proposed ambulatory actigraphy as an indirect assessment method for BPSD such as sleep disturbances, agitation, depression, and apathy. Actigraphy has been proposed to monitor specific BPSD in AD drug trials, such as sleep [53,54,55] and agitation [41,56], but, once again, has not been generalized to all drugs trials targeting these specific symptoms.

## 6. Conclusions

BPSD assessment could be improved, in clinical practice but also in clinical trials, by (1) using a clinician and a more sensitive version of the NPI in order to improve the quantitative assessment; (2) using in the inclusion criteria of studies and trials, diagnostic criteria for a specific targeted behavioral symptom; (3) using, in combination with classical assessment tools, new technologies, such as ambulatory actigraphy, in order to provide an objective assessment of behaviors in daily life.
